# Matrix Isolation of the Arsinoborene F_2_B–As=BF with an As=B Double Bond Character

**DOI:** 10.1021/acs.inorgchem.4c05418

**Published:** 2025-03-06

**Authors:** Mei Wen, Robert Medel, Pavel V. Zasimov, Sebastian Riedel

**Affiliations:** Institut für Chemie und Biochemie−Anorganische Chemie, Freie Universität Berlin, Fabeckstrasse 34/36, Berlin 14195, Germany

## Abstract

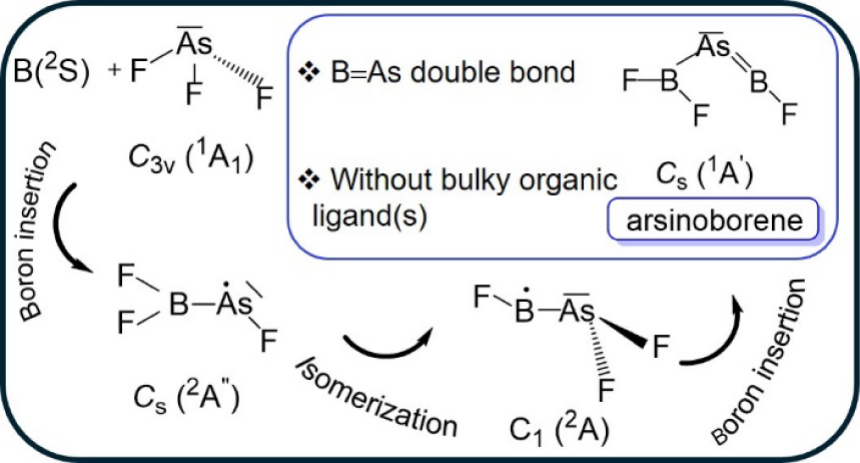

We report on the generation of F_2_B–As=BF, an arsinoborene (boranylidenearsane) with a genuine As=B double bond, where both the As and B atoms are two-coordinate. It was obtained from the reaction of AsF_3_ with laser-ablated boron atoms under cryogenic conditions in neon and argon matrices. In addition, the single-bonded arsenic–boron radicals FB–AsF_2_ and F_2_B–AsF were characterized. These species were identified by infrared spectroscopy and ^10/11^B isotope substitution in conjunction with quantum-chemical calculations at the B3LYP and CCSD(T) levels of theory. The isomerization from FB–AsF_2_ to F_2_B–AsF can be triggered by irradiation with ultraviolet light (λ = 275 nm) in argon. This discovery of the arsinoborene F_2_B–As=BF further extends the series of multiple-bonded systems between heavy main group elements and boron.

## Abstract 2

The arsinoborene F_2_B−As=BF features two-coordinate arsenic and boron atoms and a genuine As=B double bond. Additionally, the photolysis behavior of new boron−arsenic compounds upon irradiation with ultraviolet (UV) light of λ = 275 nm enabled the spectroscopic identification of FB−AsF_2_ and F_2_B−AsF.

## Introduction

Multiply bonded group 13 and group 15 element compounds are intriguing as heavy homologues to alkenes and alkynes, and as potential precursors for the fabrication of semiconductor materials.^1−5^ A variety of compounds featuring BN double bonds, such as aminoboranes (MeHN=BH_2_),^6^ and BN triple bonds, exemplified by iminoboranes^7^ have been reported. Notably, a range of the heavy analogs characterized by a BP multiple bond have been established in recent years, which are stabilized by a coordinated Lewis acid^8,9^ or base,^10−15^ a “push–pull” motif,^16^ or steric encumbrance.^17^ Besides, the linear anion [Bi≡B–B≡O]^−^, generated by laser ablation of a mixed B/Bi target, shows multiple bond character between B and Bi.^18^ Compared to the substantial advancements in boron–nitrogen and boron–phosphorus multiple bond chemistry, the exploration of species with boron–arsenic multiple bond character is relatively less developed due to the unfavorable orbital overlap and perhaps the toxicity of arsenic.^19,20^

**Scheme 1:**
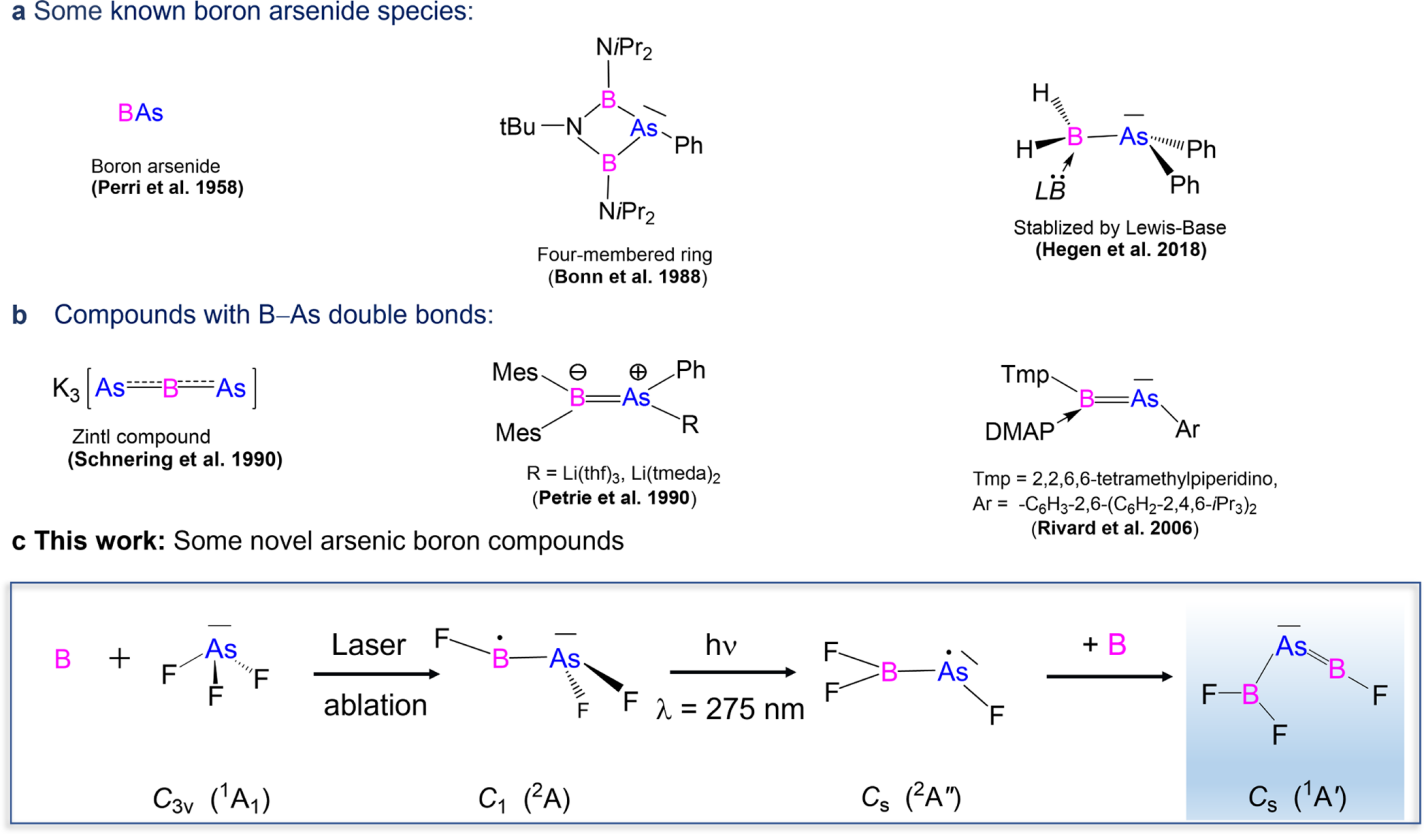
Molecules Featuring BAs Bonds Reported in Previous and in the Present Works

The first boron arsenide compound—solid, binary BAs—was prepared by combining the elements directly in vacuum-sealed quartz tubes at high temperature (Scheme 1, a).^21^ Its zincblende lattice constant of 4.777 Å corresponds to a BAs distance of 2.069 Å, close to the sum of the single bond radii^22^ of the elements, 2.06 Å. The diatomic BAs molecule is so far experimentally unexplored, with a predicted bond length of about 1.87 Å, which is in between the sum of the double^23^ and triple^24^ bond radii of 1.92 and 1.79 Å, respectively, aligning with the formal bond order of 2.5.^25^ Such short bond lengths in BAs were realized in the Zintl anions [As=B=As]^3–^ and later [P=B=As]^3–^ (1.865–1.880 Å), which were characterized to feature BAs double bonds.^26,27^ Apart from solid binary BAs species, only a few molecular compounds with a BAs bond have been reported to date, such as four-membered rings and the Lewis base (LB) stabilized arsanylborane Ph_2_As–BH_2_LB (Scheme 1a).^28,29^ Among them are some compounds which feature a B=As double bond with their structures determined by X-ray crystallography (Scheme 1b).^10,26,30^ Except for the Zintl anions mentioned above, these compounds with B–As π interaction (Scheme 1b) feature sterically demanding substituents and coordination numbers larger than two to suppress iso- and oligomerization. In recent years, bonding properties and substituent effects were computationally explored also for RAsBR′ species (termed arsinoborenes^31^ or boranylidenearsanes^10^) with two-coordinate As and B centers. It was concluded that this class of compounds shows strong multiple bond character but is thermodynamically and kinetically unstable toward isomerization reactions without bulky substituents or further coordination.^32−34^

Despite similarly pessimistic predictions for lighter phosphorus homologs, we recently reported the generation of the free two-coordinate phosphaborene F_2_B–P=BF from the reaction of laser-ablated boron with PF_3_, isolated under cryogenic conditions in neon matrices.^35^ This discovery led to the question of whether the heavier arsenic homolog could be prepared as well by applying similar strategies. Here, we report on new arsenic–boron compounds (Scheme 1c) that were identified by their infrared absorption bands, including isotopic shifts, their photochemical behavior, and by quantum-chemical calculations.

## Results and Discussion

### Assignment

**Figure 1:**
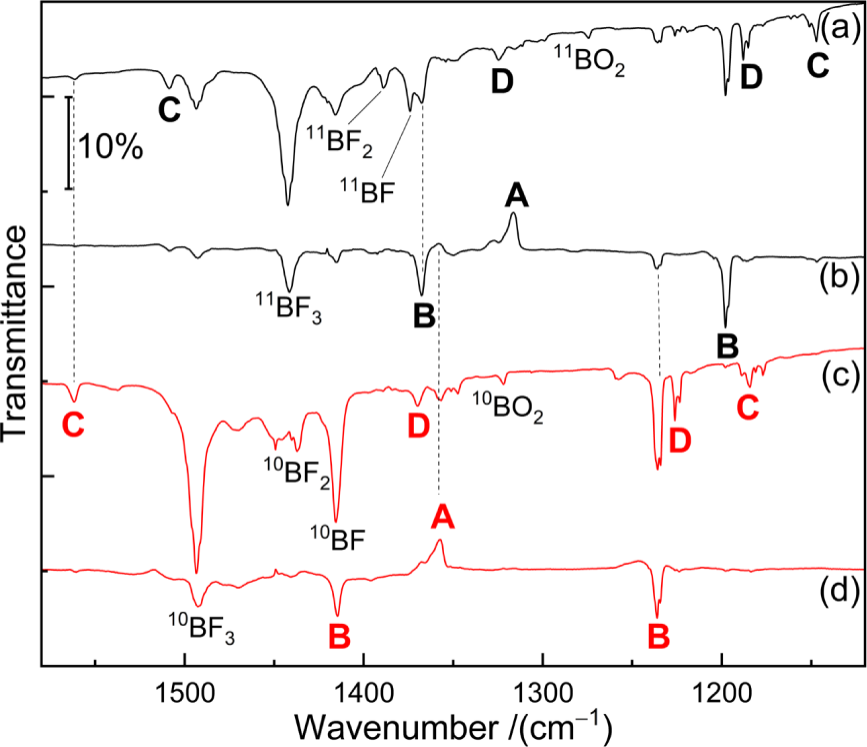
Infrared spectra obtained from codeposition of laser-ablated naturally abundant (a) or ^10^B-enriched (c) boron atoms with 0.5% AsF_3_ in solid argon, infrared difference spectra upon 15 min of 275 nm irradiation for naturally abundant (b) or ^10^B-enriched (d) boron atoms. Assignments: **A**: FB–AsF_2_, **B**: F_2_B–AsF, **C**: F_2_B–As=BF, and **D**: F_2_B–As (tentative).

Different ternary arsenic boron fluorides have been generated by codeposition of laser-ablated boron atoms with 0.05% AsF_3_ and excess neon or argon under cryogenic conditions at 5 K. Figure 1 shows the infrared spectra obtained for argon matrices. Observed but already previously reported species include BF, BF_2_, BF_3_, and BO_2_.^36−39^ New absorptions were classified into the sets **A**, **B**, **C**, and **D** based on their common intensity changes on irradiation and annealing, and assigned based on their characteristic ^10/11^B isotope shifts and comparison with quantum-chemical calculations at the CCSD(T)/def2-TZVPPD level of theory.

Considering that the lighter congener BPF_3_ was observed in our previous work,^35^ the expected initial product of this reaction is the adduct of the two reactants, BAsF_3_. Although this association is calculated to be slightly exothermic by 11.3 kJ mol^–1^ at the CCSD(T)/def2-TZVPPD level as well as barrierless, BAsF_3_ could not be identified in our experiments. Its doublet state is calculated to be 87.8 kJ mol^–1^ higher in energy than the quartet state; see Table S1 for further details. This result is qualitatively different from BPF_3_, for which the quartet state is slightly lower in energy and a substantial barrier for boron atom insertion into a P–F bond was calculated for both spin multiplicities.^35^ In contrast, there seems to be (almost) no barrier for the highly exothermic B atom insertion into an As–F bond of AsF_3_ according to the potential energy scans (Figures S2 and S3). This aligns with the observations that the adduct BAsF_3_ is absent and that the concentration of the insertion product FB–AsF_2_ increased upon annealing of the argon matrix to 20 K.
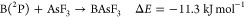
1
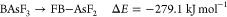
2

### FB–AsF_2_

The set of **A** bands in Figure 1 (observed only in argon) is assigned to FB–AsF_2_, the product of boron atom insertion into AsF_3_. The band position of F^10^B–AsF_2_ at 1357.7 cm^–1^ appeared blue-shifted by 41.3 cm^–1^ from F^11^B–AsF_2_, which agrees well with the value of 42.6 cm^–1^ predicted by quantum-chemical computations for the B–F vibrational stretching mode (Table 1). The symmetric and antisymmetric stretching modes of the AsF_2_ moiety, with harmonic predictions of 668.6 and 648.3 cm^–1^ (Table S2), were not identified in the spectrum. This is probably due to the overlap with the very intense absorptions of the AsF_3_ precursor in this region. Other absorption bands are predicted to have very low intensity (ca. 2 orders of magnitude lower than the ones mentioned above, Table S2). The difference spectra illustrating the effect of the 275 nm photolysis on the samples are shown in Figure 1 b,d. It is worth noting that these spectra clearly demonstrate the depletion of set **A** and the increase of set **B**. The TD-DFT calculations at the B3LYP/def2-TZVPPD level of theory of FB–AsF_2_ show a strong transition at ca. 216 nm (oscillator strength, *f* = 0.211) and a weaker transition at ca. 286 nm (*f* = 0.062) (see Figure S4) in the 200–300 nm region, which is in good agreement with its observed photolysis behavior.

**Table 1:** Observed (Ne and Ar matrices) and Calculated (CCSD(T)/def2-TZVPPD) Wavenumbers ν (in cm^–1^) and ^10/11^B isotopic shifts (Δν, cm^–1^) corresponding to the Stretching Modes of F_2_As–BF (**A**), F_2_B–AsF (**B**), F_2_B–As=BF (**C**), and F_2_B–As (**D**)

	obs. (Ne)		obs. (Ar)				
	ν (^10^B)	Δν (^10/11^B)	ν (^10^B)	Δν (^10/11^B)	cal. ν (^10^B)^d^	cal. Δν (^10/11^B)	stretching mode
**A** F_2_As–BF (*C*_1_, ^2^A)	a	a	b	b	680.3 (103)	0.0	antis. AsF_2_
	a	a	b	b	699.6 (78)	0.0	sym. AsF_2_
	a	a	1357.7	41.3	1379.2 (298)	42.6	B–F
**B** F_2_B–AsF (*C*_s_, ^2^A″)	b	b	b	b	678.1 (83)	0.0	AsF
	1240.5	39.0	1236.3	38.5	1259.6 (323)	39.6	sym. BF_2_
	1423.6	48.1	1415.1	47.3	1453.5 (295)	50.2	antis. BF_2_
**C** F_2_B–As=BF (*C*_s_,^1^A’)	1190.5	36.8	1186.5	39.4	1206.6 (518)	37.9	sym. BF_2_
	1356.5	45.2	1347.4	48.3	1390.3 (190)	47.5	antis. BF_2_
	1568.6	52.8	1562.5	53.6	1590.3 (496)	55.4	out-of-phase As=B–F
**D** F_2_B–As^c^ (*C*_2v_, ^3^A_2_)	1230.8	38.7	1224.8	38.6	1249.8 (367)	39.3	sym. BF_2_
	1379.2	45.9	1369.7	45.2	1414.7 (293)	48.0	antis. BF_2_

a Bands were not observed.

b Too weak or overlaps by broad and strong AsF_3_ bands.

c Tentative.

d Infrared intensities (in kilometers mol^–1^, parentheses) were calculated at the B3LYP/def2-TZVPPD level.

### F_2_B–AsF

The absorptions at 1236.3 and 1415.1 cm^–1^ in argon (set **B**) are assigned to F_2_^10^B–AsF on the basis of computational predictions (Table 1). These two bands correspond to the symmetric and antisymmetric stretching modes of the BF_2_ moiety, as evidenced by the boron isotope shift of 38.5 and 47.3 cm^–1^, respectively. The antisymmetric stretching mode absorption of F_2_^10^B–AsF at 1415.1 cm^–1^ coincides with the absorption of ^10^BF but nevertheless can be distinguished by its characteristic photochemical behavior (Figure 1b,d). The absorption corresponding to the As–F stretching mode of F_2_B–AsF overlaps with much more intense absorption for AsF_3_ in the spectrum, while other vibrational modes are expected to be either weak or outside the range of the detector (Table S2). Reaction (3), the anticipated rearrangement of FB–AsF_2_ to the most stable F_2_B–AsF isomer, was observed in argon upon irradiation with ultraviolet (UV) light of λ = 275 nm.
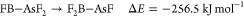
3

**Figure 2:**
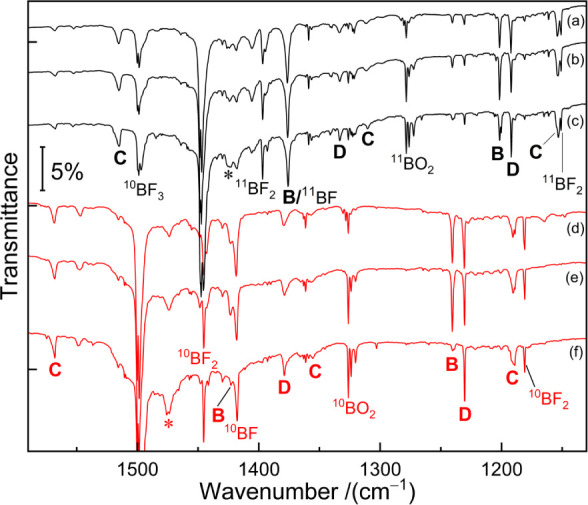
Infrared spectra of the samples obtained from codeposition of laser-ablated, naturally abundant (a–c) or ^10^B-enriched (d–f) boron atoms with 0.05% AsF_3_ in solid neon. (a,d) 60 min of sample deposition at 5 K, (b,e) 15 min of 275 nm irradiation, (c,f) annealing to 11 K. **B**: F_2_B–AsF, **C**: F_2_B–As=BF, **D**: F_2_B–As (tentative). Unknown species are marked with asterisks.

As shown in Figure 2, F_2_B–AsF absorptions at 1240.5 and 1423.6 cm^–1^ were also observed in solid neon matrices. These bands increased upon 15 min of irradiation with UV light at λ = 275 nm (Figure 2, trace b) and decreased upon further annealing to 11 K (Figure 2, trace c).

### **F**_**2**_**B**–**As=BF**

Set **C** of absorptions showed virtually no change upon irradiation or annealing (Figure S2) and was assigned to the fundamental vibrational modes of the arsinoborene F_2_B–As=BF. The strong absorption band in neon (argon) at 1190.5 (1186.5) cm^–1^ and the weaker absorption feature at 1356.5 (1347.4) cm^–1^ show large ^11^B isotopic shifts of 36.8 (39.4) and 45.2 (48.3) cm^–1^, respectively. The band positions and their associated ^11^B-isotopic shifts indicate that they are the symmetric and antisymmetric stretching modes of the BF_2_ moiety, respectively. In addition, the band at 1568.6 (1562.5) cm^–1^ (Figure 2a) in neon (argon) is attributed to the out-of-phase coupling of the As=^10^B and ^10^B–F stretches in the As=^10^B–F moiety with a large redshift of 52.8 (53.6) cm^–1^ upon ^11^B-isotope labeling (Figure 2d). The identification of F_2_B–As=BF was aided by the calculated frequencies and ^10/11^B-isotopic shifts at the CCSD(T)/def2-TZVPPD level of theory (Table 1). The symmetric and antisymmetric stretching modes of BF_2_ in the F_2_B–As=BF molecule were predicted to have 37.9 and 47.5 cm^–1 10/11^B-isotopic shifts, respectively, in good agreement with the observed ones. Also, the computed ^11^B-isotopic shift of 55.4 cm^–1^ for the out-of-phase stretching of the As=B–F moiety closely aligns with the experimentally observed value. Moreover, the infrared relative intensities calculated at the DFT level for these modes are in good agreement with the experimental results. Due to the BAsB angle being close to 90° and the heavy mass of the central As atom, the vibrations are strongly localized on either side of the molecule. Therefore, the ^10/11^B substitution on one side of the molecule is calculated to affect the vibrational wavenumbers on the other side by less than 1 cm^–1^ (Table S3). For the expected four isotopologs, only two sets of bands are resolved for this reason. Other absorption features of F_2_B–As=BF have only very weak predicted IR activity and are not observed.

Although F_2_B–As=BF was found not to decompose as a result of the λ = 275 nm photolysis, we could further verify this assignment by comparison with F_2_B–P=BF.^35^ While the error compensation regarding neglected anharmonicity is less complete than in the comparison between isotopologs, the phosphorus and arsenic compounds are similar enough that vibrational shifts are well reproduced within the harmonic approximation, as shown in Table 2 and Figure S6.

**Table 2:** Observed (Ne) and Calculated at the CCSD(T)/def2-TZVPPD Level Wavenumbers ν (in cm^–1^) and Pnictogen (Pn = P or As) Element Shifts (Δν, cm^–1^) for Modes of F_2_^10^B–As=^10^BF and F_2_^10^B–P=^10^BF.^35^ Infrared Intensities (in km mol^–1^, in parentheses) were Calculated at the B3LYP/def2-TZVPPD Level

	cal. ν (Pn = P)	obs. ν (Pn = P)	cal. ν (Pn = As)	obs. ν (Pn = As)	Δν (cal.)	Δν (exp.)
F_2_B–Pn=BF (*C*_s_, ^1^A’)	1218.4 (569)	1205.4	1206.6 (518)	1190.5	11.8	14.9
	1387.2 (217)	1354.3	1390.3 (190)	1356.5	–3.1	–2.2
	1636.3 (548)	1613.1	1590.3 (496)	1568.6	46.0	44.5

### **F**_**2**_**B**–**As**

The observed absorptions at 1230.8 (1224.8) and 1379.2 (1369.7) cm^–1^ in neon (argon) for set **D** are close to the absorptions for sets **B** and **C** (assigned to F_2_B–AsF and F_2_B–As=BF) with similar boron isotopic shifts, which indicates that the responsible species has a BF_2_ moiety as well. Set **D** showed no obvious changes upon subsequent photolysis in the range of 730–220 nm or annealing. By comparison with the computed infrared vibrations and boron isotopic shifts for all possible candidates, this set is tentatively assigned to triplet arsinidene F_2_B–As (Table 1). Furthermore, the TD-B3LYP/def2-TZVPPD computation of F_2_B–As showed that it starts to absorb at ca. 250 nm, which is below the wavelength photolysis region used in this work (see Figure S5). We note that triplet F_2_B–As would be expected to react with doublet B atoms on annealing, which is not observed. This might possibly be attributed to the competition with more abundant reaction partners of B, e.g., AsF_3_ and radical species such as B, F, and BF_2_.

### Theoretical Characterization

**Figure 3:**
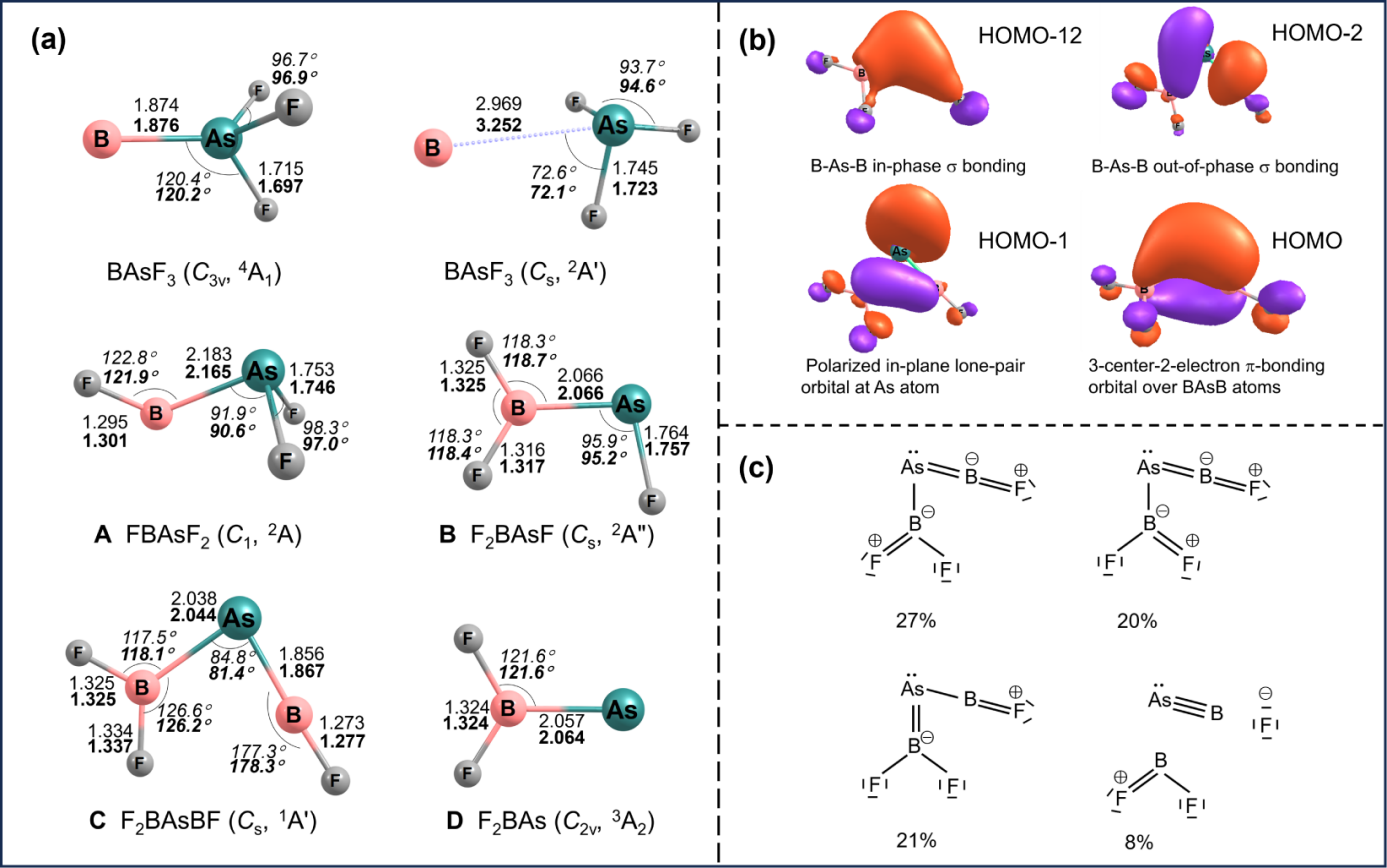
(a) The structures of BAsF_3_ (not observed), FB–AsF_2_ (**A**), F_2_B–AsF (**B**), F_2_B–As=BF (**C**), and F_2_B–As (**D**) optimized at the B3LYP/def2-TZVPPD (upper values) and CCSD(T)/def2-TZVPPD (lower, bold values) methods. Bond lengths (Å), angles (deg), and molecular symmetries are also listed; (b) selected bonding Kohn–Sham molecular orbitals of F_2_B–As=BF calculated at the B3LYP/def2-TZVPPD level; (c) major Lewis resonance structures of F_2_B–As=BF as predicted by natural resonance theory (NRT).

The equilibrium structures of the compounds assigned to sets **A**, **B**, **C**, and **D** are illustrated in Figure 3.

The species FB–AsF_2_ (**A**, only observed in argon) has a doublet ground state with *C*_1_ symmetry, with the spin density mainly located at the boron atom (Figure S8). The B–As bond length of 2.165 Å at the CCSD(T) level is slightly longer than the sum of the single bond radii of the elements, 2.06 Å,^22^ in agreement with the Wiberg bond index of 0.93 being slightly lower than unity.

The species F_2_B–AsF (**B**) possesses *C*_s_ symmetry, and the spin density is localized at the arsenic atom (Figure S8). The calculated B–As bond length of 2.066 Å and the Wiberg bond index of 1.06 both indicate a slightly stronger bond than in species **A**. For the intramolecular isomerization from FB–AsF_2_ to F_2_B–AsF the calculations confirm that this process via F-atom shift is highly exothermic with a relatively low activation barrier of 37.2 kJ mol^–1^ (Figures S10 and Figure S11).

The species F_2_B–As=BF (**C**) is predicted to have a closed-shell singlet ground state with *C*_s_ symmetry. Its molecular structure closely matches that of its phosphorus counterpart F_2_B–P=BF. The calculated B–As bond length of 1.867 Å in the As=B–F moiety of F_2_B–As=BF is between the sum of the double^23^ and triple^24^ bond radii of 1.92 and 1.79 Å, respectively, and is among the shortest reported for an experimentally observed species, similar to the Zintl anions [As=B=As]^3–^ and [P=B=As]^3–^ (1.865–1.880 Å).^26,27^ The Wiberg bond index of 1.757 indicates substantial As=B double bond character. The B–As bond length in the F_2_B–As moiety is computed as 2.044 Å with a Wiberg bond index of 1.062, both typical for a single bond.^22^ The Kohn–Sham molecular orbitals (MOs) of F_2_B–As=BF are illustrated in Figure 3 b. The MOs represent in- and out-of-phase B–As=B σ-bonding. In addition, there is a nonbonding lone pair orbital that lies in the B–As=B plane and the As–B(1) π-bond orthogonal to the B–As=B plane that is partly delocalized over this moiety. Noteworthy is the unusual acute BAsB bond angle of 81.4° (84.8°) at the CCSD(T) (B3LYP) level, similar to the BPB angle in the analogous phosphaborene F_2_B–P=BF, which we attribute to the presence of a weak B···B attraction.^35^

The electronic structure of F_2_B–As=BF can be described by the major Lewis resonance structures rationalized by natural resonance theory (NRT) analysis (Figure 3). The NRT As=B bond order in the As=B–F moiety is 1.887. The high covalent (1.37) and low ionic (0.51) contributions to the As=B bond imply that this bond is mostly covalent. The B–F bond in this moiety is highly polar, as evidenced by the low covalent (0.36) and high ionic (1.36) contributions to the total Natural Bond Order (NBO) of 1.72. The polarity of the B–F bond is also reflected in the high opposite NPA charges of B (+0.68) and F (−0.46). F_2_B–As=BF is the thermodynamically most stable isomer, trailed by the As–B(F)(BF_2_) isomer by about 78.9 kJ mol^–1^ at the B3LYP level (Figure S9). This energy advantage is far bigger than for any other arsinoborene computationally explored so far,^32,33^ likely the result of the combination of a π-accepting (F_2_B−) and a π-donating substituent (−F), as it was proposed for reported phosphaborenes with two-coordinate phosphorus and boron atoms.^16,35^

**Scheme 2:**
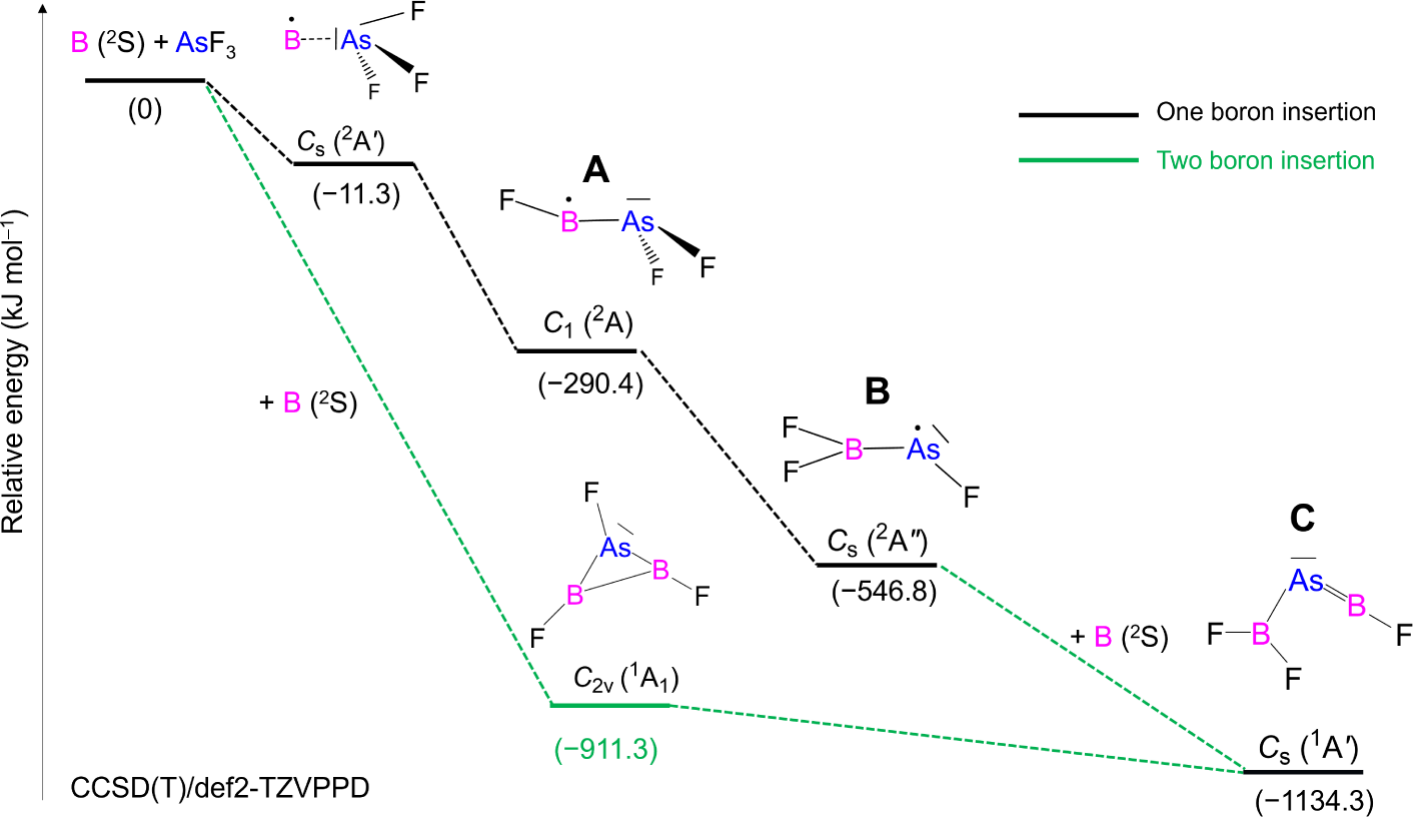
Relative Zero-Point Corrected Energies in kJ mol^–1^ for Species Formed from Laser-Ablated Boron Atoms with AsF_3_ Computed at the CCSD(T)/def2-TZVPPD Level (Distances are not to Scale)

The most plausible method of FB–AsF_2_ formation is the (almost) barrierless insertion of one boron atom into an As–F bond of AsF_3_. After that, FB–AsF_2_ may isomerize to the most thermodynamically stable F_2_B–AsF isomer via fluorine transfer. This transfer may be induced by excess energy released in the insertion reaction or triggered by irradiation with UV light of λ = 275 nm (Scheme 2). As shown in Scheme 2, we suggest that two possible pathways may lead to the generation of compound F_2_B–As=BF. First, this species might be formed via the reaction of two boron atoms (or one B_2_ molecule) with one AsF_3_ molecule through an unobserved cyclic FB–(AsF)–BF intermediate followed by isomerization (the corresponding rearrangement is predicted at the CCSD(T) level to be highly exothermic by 223.0 kJ mol^–1^). Second, it might be produced via boron atom insertion into the As–F bond of F_2_B–AsF (Scheme 2). Because the intermediates FB–AsF_2_ and F_2_B–AsF are observed but FB–(AsF)–BF is not, the second pathway appears more likely.

F_2_B–As (**D**) is the thermodynamically most stable isomer of its formula according to the DFT computations^32^ and has a triplet ^3^A_2_ electronic ground state with *C*_2v_ symmetry, as shown in Figure 3, with the two unpaired electrons located at the As atom (Figure S8), similar to previously reported matrix-isolated organic arsinidenes.^40,41^ The B–As bond length in F_2_B–As (**D**) is nearly identical to the one in species F_2_B–AsF (**B**). It might be formed by the reaction of a boron atom with an AsF_2_ radical fragment or by fluorine atom loss or intermolecular transfer from F_2_B–AsF, e.g., to a boron atom (BF, BF_2_, and BF_3_ are observed).

## Conclusion

In summary, we report the formation of arsenic boron compounds FB–AsF_2_, F_2_B–AsF, F_2_B–As=BF, and tentatively F_2_B–As. These new species were created by the reaction of laser-ablated boron atoms with AsF_3_, trapped in neon and argon matrices, identified by infrared spectroscopy, and further characterized by DFT and CCSD(T) calculations. It is found that boron atom insertion into a bond of AsF_3_ occurs barrierlessly to form FB–AsF_2_ (only observed in argon), which further rearranges exothermically to the lower energy isomer F_2_B–AsF by irradiation at 275 nm. A second boron atom insertion leads to F_2_B–As=BF, which is an arsinoborene with an authentic As=B double bond and is thermodynamically and photochemically stable against isomerization.

## Experimental and Computational Section

### Experimental Section

The experimental method used for the laser ablation of boron atoms for matrix isolation infrared spectroscopy has been described in more detail in our previous work.^42^

Briefly, the 1064 nm fundamental of a Nd:YAG laser (Continuum, Minilite II, 10 Hz repetition rate) with a pulse energy of 50–65 mJ per 10 ns pulse was focused onto the rotating bulk boron target to produce boron atoms through a hole in the cold mirror. Natural abundance boron (^10^B, 19.8%; ^11^B, 80.2%) and ^10^B-enriched (>95%) targets were used in different experiments. The evaporated boron atoms with an energetic plasma beam reacting with 0.05% AsF_3_ (99%) in excess neon (99.999%, Air Liquide) or argon (99.999%, Sauerstoffwerk Friedrichshafen) were deposited onto a cryogenic gold-plated copper mirror at 5 K by mounting it on a closed-cycle helium cryostat (Sumitomo Heavy Industries, RDK-205D) inside a vacuum chamber. AsF_3_ was prepared by reacting concentrated sulfuric acid with a mixture of arsenic trioxide and calcium fluoride and then distilled: As_2_O_3_ + 3 CaF_2_ + 3 H_2_SO_4_ → 2 AsF_3_ + 3 CaSO_4_ + 3 H_2_O.^43^ FTIR spectra were recorded on a Bruker Vertex 80v spectrometer at 0.5 cm^–1^ resolution in the region between 4000 and 450 cm^–1^ by using a liquid-nitrogen-cooled mercury cadmium telluride (MCT) detector. The matrix samples were annealed at different temperatures and irradiated by ultraviolet (UV) light using a UV LED (275 nm, Avonec 6868, 10–15 mW).

### Theoretical Section

Quantum chemical calculations with density functional theory (DFT) were performed using the *Gaussian* 16 program revision A.03 package^44^ employing the hybrid functional B3LYP.^45−48^ The coupled-cluster singles-doubles with perturbative triples excitations (CCSD(T)) calculations^49,50^ based on the RHF reference wavefunction (RHF-UCCSD(T) or RHF-CCSD(T)) were carried out in the Molpro 2019.1.0 software package.^51^ Frequency calculations were carried out analytically for the B3LYP method and numerically for the CCSD(T) method. The transition states connecting both minima and the corresponding intrinsic reaction coordinate (IRC) calculation were performed at the B3LYP level.

For all calculations, the def2-TZVPPD basis set was used for B, F, and As atoms.^52^ All energies are provided in this work, including harmonic zero-point energy corrections. The NBO and natural resonance theory (NRT) analyses were carried out at the B3LYP/def2-TZVPPD level using the NBO 7.0 program.^53^ The Kohn–Sham molecular orbitals were visualized using the program Chemcraft version 1.8.^54^

## Supporting Information Available

IR spectra obtained from codeposition of boron atoms with AsF_3_ in argon; calculated wavenumbers and ^10/11^B isotopic shifts for assigned compounds; calculated structural parameters of isomers and transition states; and Cartesian coordinates (PDF)
