# Supplementary material for: Matrix Isolation of the Arsinoborene F2B–As=BF with an As=B Double Bond Character
Source: Inorg Chem. 2025 Mar 6;64(10):5105–12. doi: 10.1021/acs.inorgchem.4c05418 (PMC11920953; doi:10.1021/acs.inorgchem.4c05418)
Supplement: Supplementary file 1 — ic4c05418_si_001.pdf [file ic4c05418_si_001.pdf]

Supporting Information

Matrix Isolation of the Arsinoborene F_2B–As=BF with an As=B Double Bond Character

Mei Wen, Robert Medel, Pavel V. Zasimov, Sebastian Riedel*

Freie Universität Berlin, Institut für Chemie und Biochemie–Anorganische Chemie, Fabeckstrasse 34/36, 14195 Berlin, Germany. E-Mail: s.riedel@fu-berlin.de

Contents

Figure S1. IR obtained from codeposition of laser-ablated naturally abundant (a) or ^{10}B-enriched (d) boron atoms with 0.5% AsF_3 in Ar……….......…………….......................…3
Figure S2. IR obtained from codeposition of laser-ablated naturally abundant (a) or ^{10}B-enriched (d) boron atoms with 0.05% AsF_3 in Ne……….......…………….......................…4

Table S1. Calculated stretching wavenumbers ν (in cm^{-1}) and $^{10/11}$B isotopic shifts (Δν, cm^{-1}) at the B3LYP and CCSD(T) levels of $BAsF_3$ (C_{3v}, $^4A_1$) and $BAsF_3$ (C_s, $^2A'$) ……….................5

Figure S3. The calculated PES scan along B…As coordinate at the B3LYP/def2-TZVPPD level in the B…AsF_3 molecule for the reaction from B + AsF_3 to B…AsF_3….......……………....5

Figure S4. The calculated PES scan along B…F coordinate at the B3LYP/def2-TZVPPD level in the B…AsF_3 molecule for the reaction from B…AsF_3 to FB−AsF_2.........……………........6

Table S2. Calculated stretching wavenumbers ν (in cm^{-1}) and $^{10/11}$B isotopic shifts (Δν, cm^{-1}) at the B3LYP/def2-TZVPPD level of compounds **A**, **B, C** and **D** ……….......……………7

Figure S5. TD-DFT spectrum of compound **A** computed at B3LYP/def2-TZVPPD………...8

Figure S6. TD-DFT spectrum of compound **D** computed at B3LYP/def2-TZVPPD…………8

Table S3 Observed and Calculated fundamental frequencies (cm^{-1}) of F_2BAsBF (C_s, $^1A'$) in neon matrix at B3LYP/def2-TZVPPD and CCSD(T)/def2-TZVPPD level.………………..........9

Figure S7. Infrared spectra obtained from codeposition of laser-ablated naturally abundant boron atoms with 0.05% AsF_3 or 0.05% PF_3 in solid neon...………..10

Figure S8. Computed spin-density iso-surfaces of $FBAsF_2$, F_2BAsF and F_2BAs at 0.03 Å^{-3}, calculated at the B3LYP/def2-TZVPPD level of theory.…………….....................................11

Figure S9. Isomers of the formula B_2AsF_3 with relative stabilities (electronic energies + ZPE correction) in kJ mol^{-1} calculated at B3LYP/def2-TZVPPD level of theory...............11

Figure S10. Transition state structure (TS1-D) for the doublet hypersurface of Scheme 2 and its frequencies (cm^{-1}) calculated at the B3LYP/def2-TZVPPD level of theory……………......12

Figure S11. Intrinsic reaction coordinate (IRC) via the transition state TS2-D (Scheme S1) for the mutual isomerization of doublet FBAsF2 and doublet F_2BAsF obtained at the B3LYP/def2-TZVPPD level of theory……………………………………………….........12

Figure S12. Transition state structure (TS2) for the doublet hypersurface of Scheme 2 and its frequencies (cm^{-1}) calculated at the B3LYP/def2-TZVPPD level of theory………………........13

Figure S13. Intrinsic reaction coordinate (IRC) via the transition state TS2 (Scheme 2) for the mutual isomerization of doublet cycle-FBAsFBF and doublet F_2B−As=BF obtained at the B3LYP/def2-TZVPPD level of theory…………………........………........………..........13

Scheme S1. Potential energy surface (electronic energies + ZPE correction) in kJ mol^{-1} for species formed from laser ablated boron atoms with AsF_3 obtained at the B3LYP/def2-TZVPPD level of theory…………………........………........………..................................14

Calculated atomic coordinates of compounds **A, B, C and D**……….………………………....15

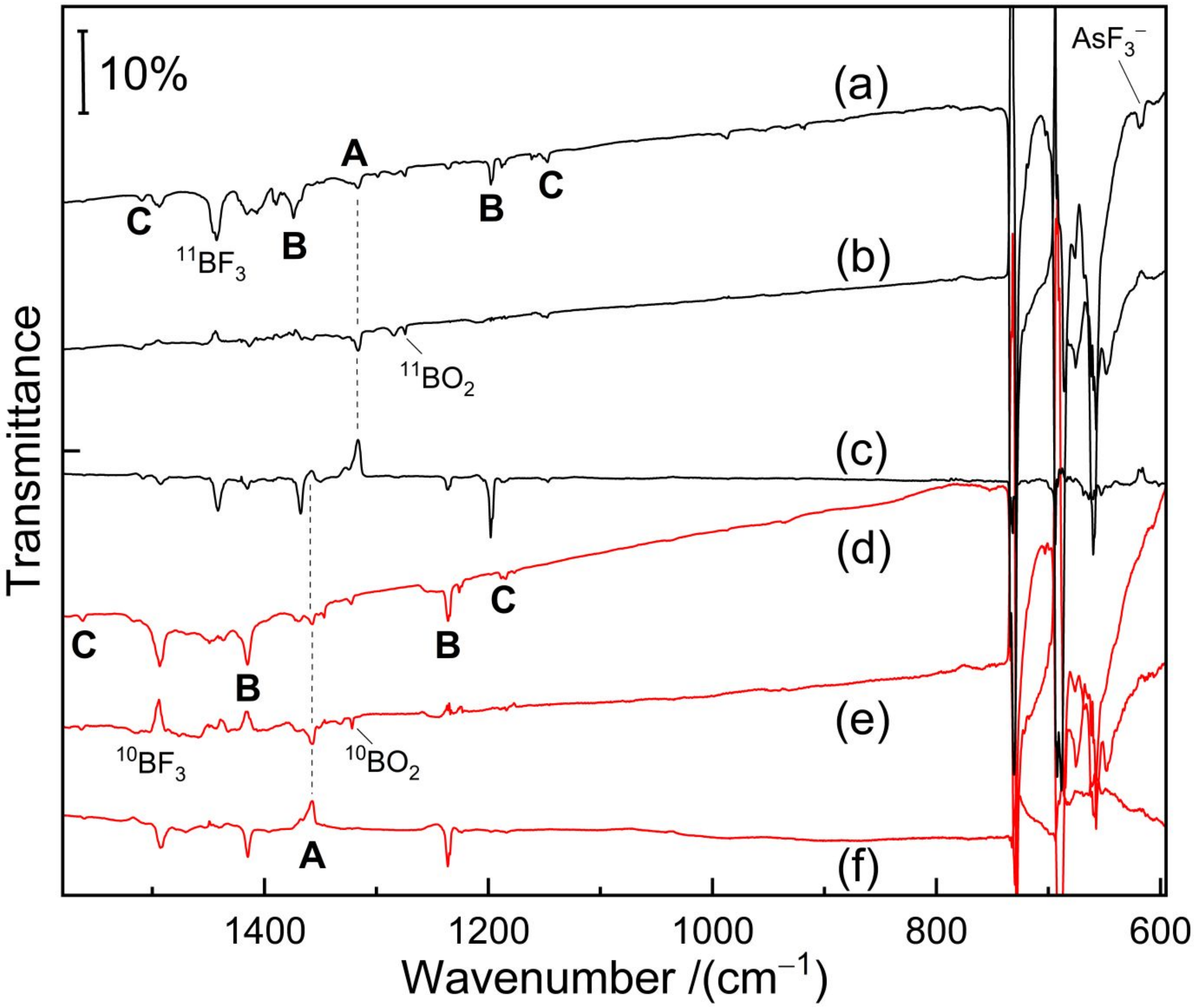

Figure S1. Infrared spectra obtained from codeposition of laser-ablated naturally abundant (a) or ^{10}B-enriched (d) boron atoms with 0.5% AsF_3 in solid argon, (b) infrared difference spectra after annealing to 20 K for naturally abundant boron atoms, (c) infrared difference spectra upon 15 min of 275 nm irradiation for naturally abundant boron atoms, , (e) infrared difference spectra after annealing to 20 K for ^{10}B-enriched boron atoms, (f) IR difference spectra upon 15 min of 275 nm irradiation for ^{10}B-enriched boron atoms. **A**: FB−AsF_2, **B**: F_2B−AsF, **C**: F_2B−As=BF.

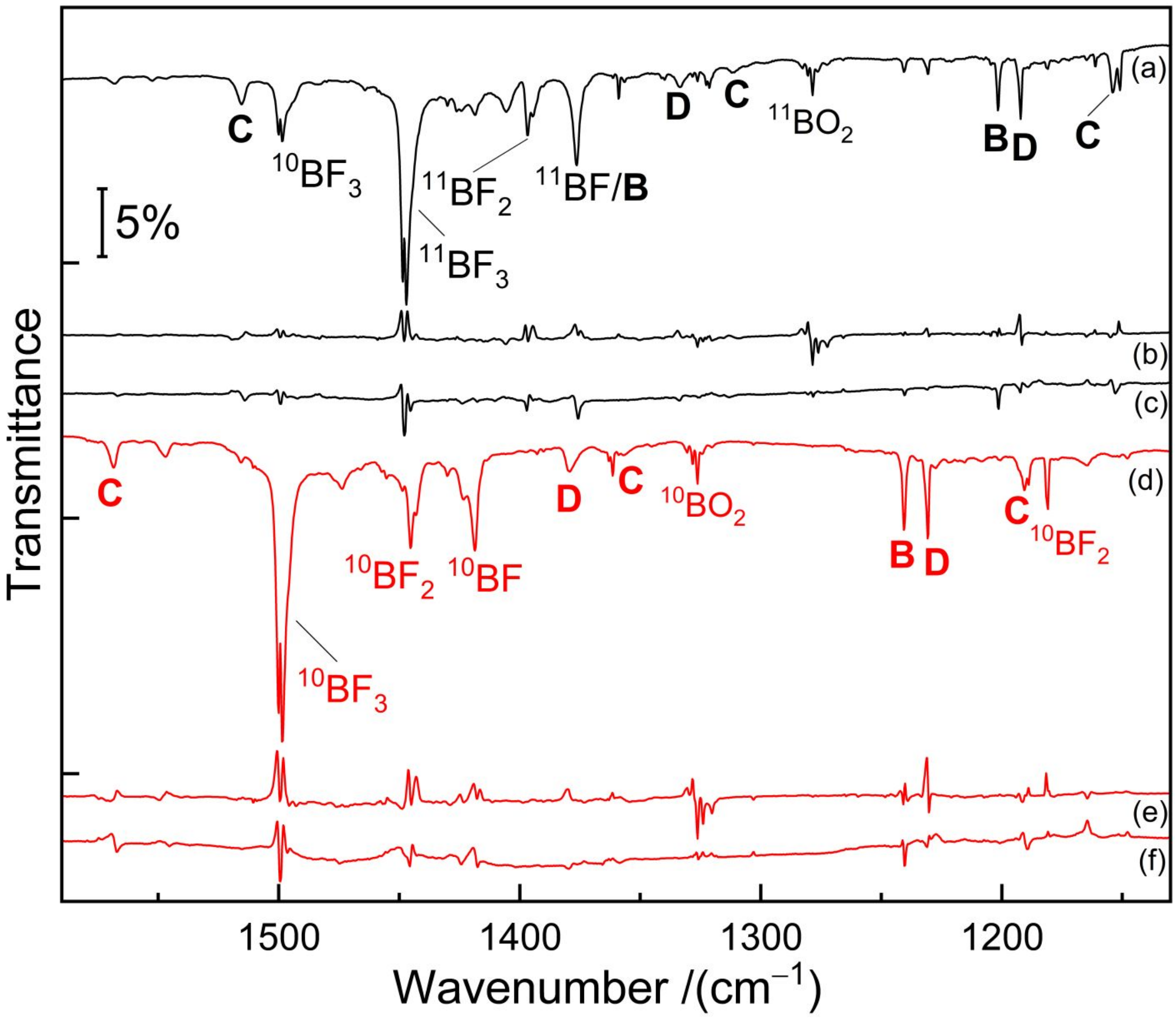

Figure S2. Infrared spectra of the samples obtained from co-deposition of laser-ablated, naturally abundant (a), (b), (c) or ^{10}B-enriched (d), (e), (f) boron atoms with 0.05% AsF_3 in solid neon. (a, d) 60 min of sample deposition at 5 K, (b, e) difference spectra after annealing to 9 K, (c, f) difference spectra upon 15 min of 275 nm irradiation. Assignments: **B:** $F_2B{-}AsF$, **C:** $F_2B{-}As{=}BF$, and **D:** $F_2B{-}As$ (tentative).

Table S1. Non-scaled harmonic wavenumbers ν (in cm^{-1}), $^{10/11}B$ isotopic shifts ($\Delta\nu$, cm^{-1}), and infrared intensities (in km mol^{-1}, in parentheses) calculated at the B3LYP/def2-TZVPPD and CCSD(T)/def2-TZVPPD levels for the $BAsF_3$ (C_{3v}, $^4A_1$) and $BAsF_3$ (C_s, $^2A'$) species.

	B3LYP		CCSD(T)	
	$\nu(^{10}B)$	$\Delta\nu(^{10/11}B)$	$\nu(^{10}B)$	$\Delta\nu(^{10/11}B)$
$BAsF_3$ (C_{3v}, $^4A_1$)	217.1 (1×2)	4.8	232.5	4.9
	261.3 (9×2)	1.5	281.8	1.9
	326.5 (43)	0.5	342.1	0.6
	691.5 (97)	7.1	734.0	9.5
	696.0 (105×2)	0.0	747.4	0.0
	820.7 (62)	25.7	850.3	24.6
$BAsF_3$ (C_s, $^2A'$)	29.7 (0)	0.8	62.4	0.2
	61.3 (1)	1.8	84.4	0.5
	114.2 (14)	3.9	120.6	3.8
	232.4 (20)	0.1	248.6	0.1
	251.7 (3)	0.0	269.1	0.0
	323.5 (25)	0.1	339.7	0.1
	639.7 (188)	0.0	647.7	0.0
	647.3 (116)	0.0	655.1	0.0
	681.5 (88)	0.1	688.8	0.2

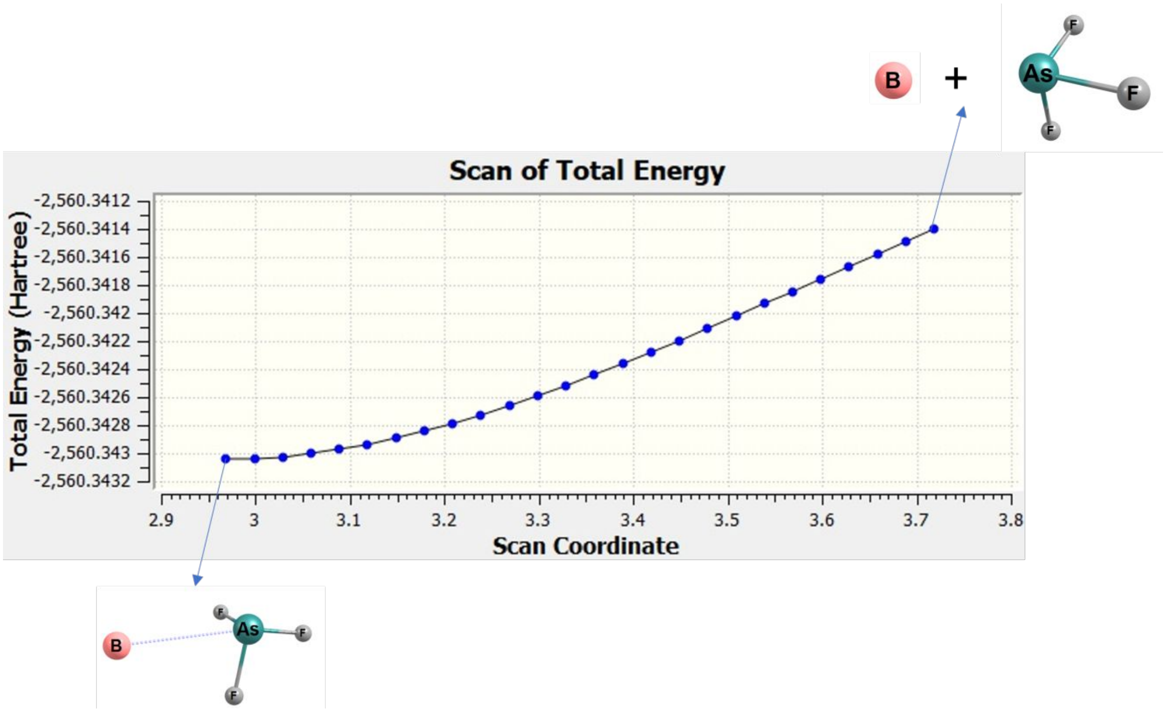

Figure S3. Calculated Potential Energy Surface scan along the B…As coordinate at the B3LYP/def2-TZVPPD level for the reaction from B + AsF_3 to B···AsF_3.

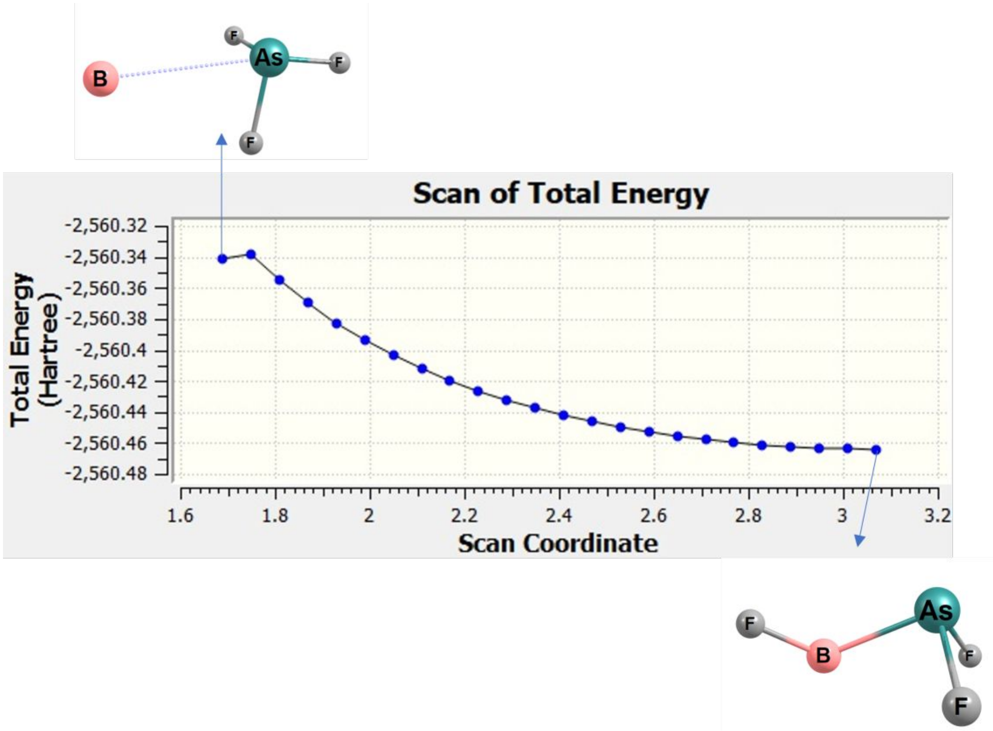

Figure S4. Calculated Potential Energy Surface scan along B…F coordinate at the B3LYP/def2-TZVPPD level for the reaction from B···AsF_3 to FB–AsF_2.

Table S2. Non-scaled harmonic wavenumbers ν (in cm^{-1}), $^{10/11}B$ isotopic shifts ($\Delta\nu$, cm^{-1}), and infrared intensities (in km mol^{-1}, in parentheses) calculated at the B3LYP/def2-TZVPPD level for the $FB{-}AsF_2$ (**A**), $F_2B{-}AsF$ (**B**), $F_2B{-}As{=}BF$ (**C**), and $F_2B{-}As$ (**D**) species.

	$\nu(^{10}B)$	$\nu(^{11}B)$	$\Delta\nu(^{10/11}B)$	stretching mode
**A** F_2AsBF	27.4 (1)	26.6 (1)	0.8	
(C_1, ^2A)	149.6 (3)	149.3 (3)	0.3	
	187.0 (2)	183.4 (2)	3.6	
	238.7 (4)	236.5 (4)	2.2	
	277.3 (4)	275.9 (4)	1.4	
	494.8 (3)	476.3 (3)	18.5	B–As
	648.3 (103)	648.3 (103)	0.0	antis. AsF_2
	668.6 (79)	668.5 (79)	0.1	sym. AsF_2
	1372.6 (298)	1330.6 (280)	42.0	B–F
**B** F_2BAsF	70.3 (2)	70.3 (2)	0.0	
(C_s, $^2A''$)	129.7 (3)	129.6 (3)	0.1	
	267.6 (1)	266.4 (1)	1.2	
	312.6 (13)	311.6 (13)	1.0	B–As
	479.3 (27)	460.1 (25)	19.2	
	596.0 (17)	589.9 (18)	6.1	
	656.3 (84)	656.3 (84)	0.0	As–F
	1232.4 (374)	1194.1 (345)	38.3	sym. BF_2
	1422.6 (311)	1373.7 (290)	48.9	antis. BF_2
**C** F_2BAsBF	46.6 (0)	46.5 (0)	0.1	
(C_s, $^1A'$)	65.6 (1)	65.4 (1)	0.2	
	249.3 (1)	247.1 (1)	2.2	
	310.8 (4)	310.1 (4)	0.7	B–As
	374.5 (2)	360.1 (1)	14.4	
	435.1 (8)	419.7 (7)	15.4	
	514.8 (2)	509.2 (1)	5.6	As–B
	537.3 (13)	515.7 (12)	21.6	
	593.1 (34)	587.1 (37)	6.0	
	1189.9 (565)	1153.0 (518)	36.9	sym. BF_2
	1365.5 (201)	1319.0 (190)	46.5	antis. BF_2
	1586.5 (534)	1531.6 (497)	54.9	out-of-phase As=BF
**D** F_2BAs	214.9 (1)	213.5 (1)	1.4	
(C_{2v}, $^3A_2$)	300.4 (8)	299.9 (7)	0.5	B–As
	487.3 (26)	467.9 (24)	19.4	
	595.5 (19)	589.4 (21)	6.1	
	1224.1 (398)	1186.0 (367)	38.1	sym. BF_2
	1380.0 (315)	1333.4 (293.4)	46.6	antis. BF_2

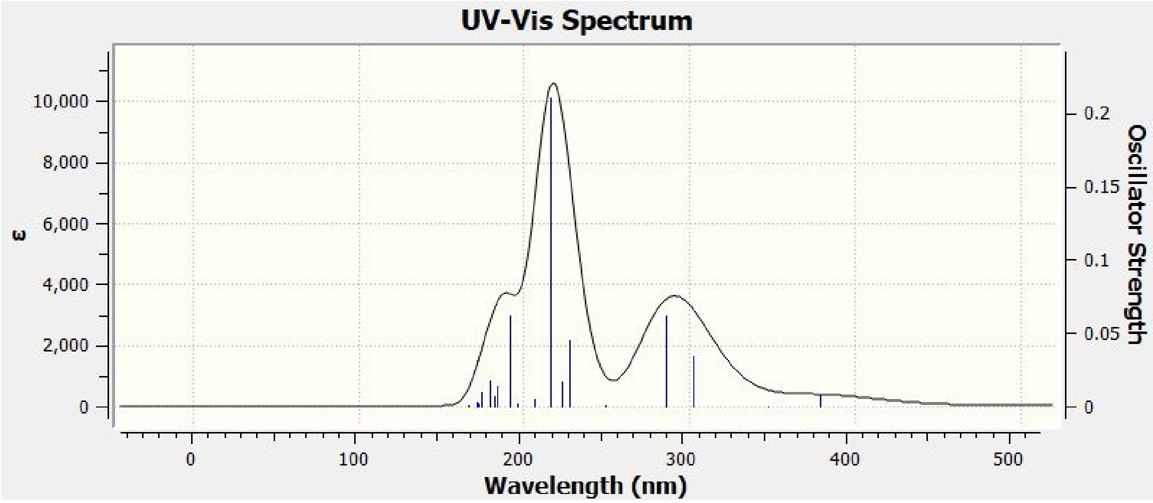

Figure S5. TD-DFT spectrum of compound **A** (FB−AsF_2) computed at the B3LYP/def2-TZVPPD level.

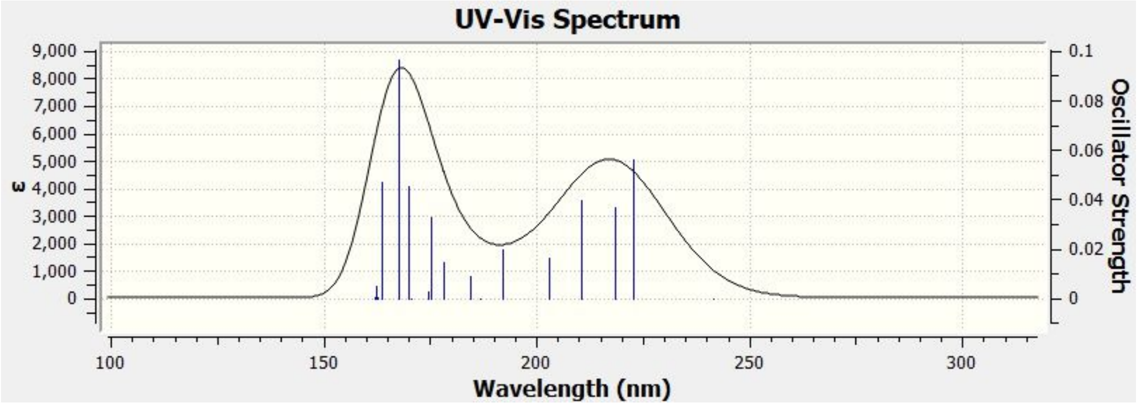

Figure S6. TD-DFT spectrum of compound **D** (F_2B−As) computed at the B3LYP/def2-TZVPPD level.

**Table S3** Harmonic wavenumbers (cm^{-1}) of $^{10/11}B$ isotopologs of **C** $F_2B–As=BF$ (C_s, $^1A'$) calculated at B3LYP/def2-TZVPPD and CCSD(T)/def2-TZVPPD levels and the absorption features observed in neon and argon matrices.				
Approximate mode description	B3LYP	CCSD(T)	Obs. (neon)	Obs. (argon)
$F_2{}^{11}BAs^{11}BF$				
out-of-phase $As={}^{11}BF$	1531.6 (496)	1534.9	1515.8	1508.9
antis. $^{11}BF_2$	1319.0 (190)	1342.8	1311.3	1299.1
sym. $^{11}BF_2$	1153.0 (518)	1168.7	1153.7	1147.1
$^{10}B–As$ stretch	587.1 (37)	591.8	n.o.	n.o.
$^{10}B–As$ twist	515.7 (12)	524.0	n.o.	n.o.
$As–{}^{11}B$ stretch	509.2 (1)	509.1	n.o.	n.o.
$As={}^{11}BF$ bend	419.7 (7)	417.7	n.o.	n.o.
$As={}^{11}BF$ bend	360.1 (1)	355.6	n.o.	n.o.
$^{10}B–As$ stretch	310.1 (4)	315.8	n.o.	n.o.
$^{10}B–As$ twist	247.1 (1)	247.2	n.o.	n.o.
$^{10}B–As–{}^{11}B$ scissor	65.4 (1)	73.3	n.o.	n.o.
$^{10}BF_2$ twist	46.5 (0)	45.0	n.o.	n.o.
$F_2{}^{11}BAs^{10}BF$				
out-of-phase $As={}^{10}BF$	1586.0 (532)	1589.6	1568.6	1562.5
antis. $^{11}BF_2$	1319.1 (193)	1343.0	1311.3	1299.1
sym. $^{11}BF_2$	1153.3 (516)	1169.1	1153.7	1147.1
$^{11}B–As$ stretch	587.1 (37)	591.9	n.o.	n.o.
$^{11}B–As$ twist	516.1 (12)	524.3	n.o.	n.o.
$As–{}^{10}B$ stretch	514.7 (2)	513.9	n.o.	n.o.
$As={}^{10}BF$ bend	435.0 (8)	433.6	n.o.	n.o.
$As={}^{10}BF$ bend	374.3 (2)	369.8	n.o.	n.o.
$^{11}B–As$ stretch	310.2 (4)	315.9	n.o.	n.o.
$^{11}B–As$ twist	247.6 (1)	247.6	n.o.	n.o.
$^{11}B–As–{}^{10}B$ scissor	65.5 (1)	73.4	n.o.	n.o.
$^{11}BF_2$ twist	46.6 (0)	45.0	n.o.	n.o.
$F_2{}^{10}BAs^{11}BF$				
out-of-phase $As={}^{11}BF$	1532.3 (500)	1535.7	1515.8	1508.9
antis. $^{10}BF_2$	1365.3 (196)	1390.0	1356.5	1347.4
sym. $^{10}BF_2$	1189.5 (567)	1206.1	1190.5	1186.5
$^{10}B–As$ stretch	593.1 (34)	597.8	n.o.	n.o.
$^{10}B–As$ twist	537.1 (13)	545.8	n.o.	n.o.
$As–{}^{11}B$ stretch	509.3 (1)	509.2	n.o.	n.o.
$As={}^{11}BF$ bend	419.8 (8)	417.8	n.o.	n.o.
$As={}^{11}BF$ bend	360.2 (1)	355.7	n.o.	n.o.
$^{10}B–As$ stretch	310.7 (4)	316.4	n.o.	n.o.
$^{10}B–As$ twist	248.8 (1)	248.9	n.o.	n.o.
$^{10}B–As–{}^{11}B$ scissor	65.5 (1)	73.3	n.o.	n.o.
$^{10}BF_2$ twist	46.6 (0)	45.0	n.o.	n.o.
$F_2{}^{10}BAs^{10}BF$				
out-of-phase $As={}^{10}BF$	1586.5 (534)	1590.3	1568.6	1562.5
antis. $^{10}BF_2$	1365.5 (202)	1390.3	1356.5	1347.4
sym. $^{10}BF_2$	1189.9 (565)	1206.6	1190.5	1186.5

^{10}B–As stretch	593.1 (34)	597.8	n.o.	n.o.
^{10}B–As twist	537.3 (13)	546.0	n.o.	n.o.
As–^{10}B stretch	514.8 (2)	514.0	n.o.	n.o.
As=^{10}BF bend	435.1 (8)	433.7	n.o.	n.o.
As=^{10}BF bend	374.5 (2)	369.9	n.o.	n.o.
^{10}B–As stretch	310.8 (4)	316.4	n.o.	n.o.
^{10}B–As twist	249.3 (1)	249.4	n.o.	n.o.
^{10}B–As–^{11}B scissor	65.6 (1)	73.5	n.o.	n.o.
$^{10}BF_2$ twist	46.6 (0)	45.0	n.o.	n.o.

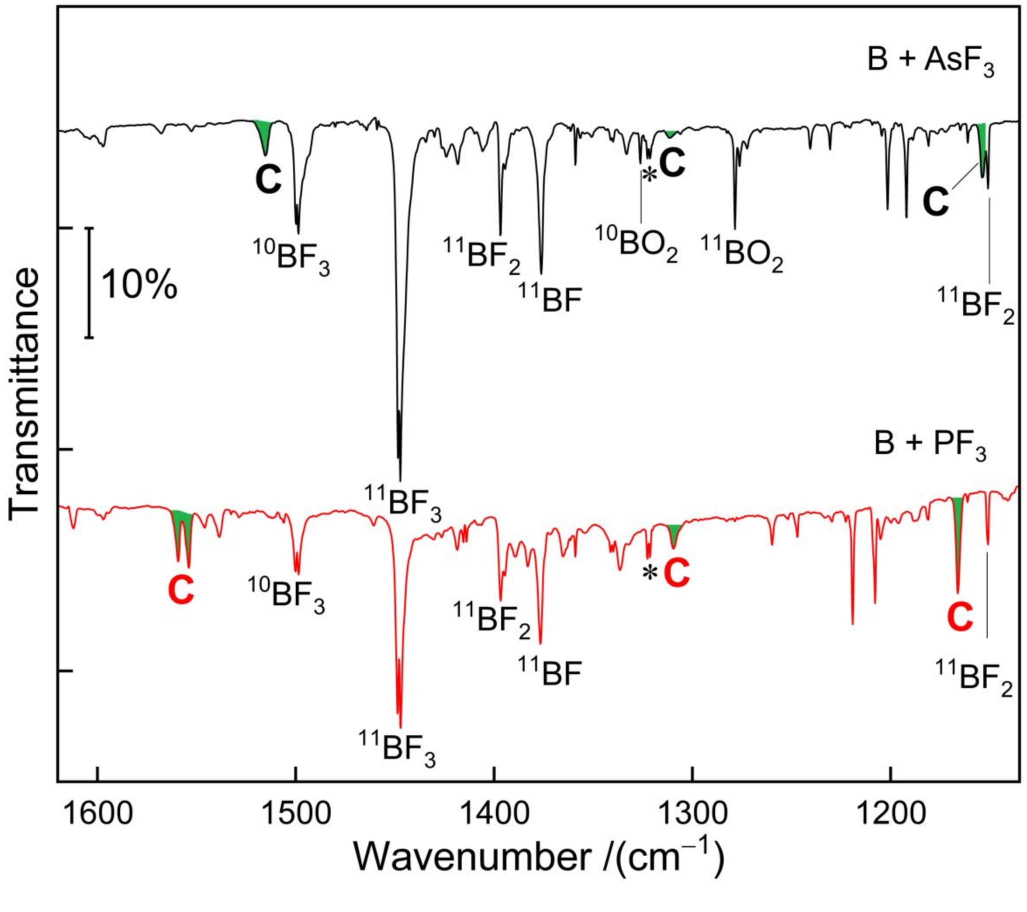

Figure S7. Infrared spectra obtained from codeposition of laser-ablated naturally abundant boron atoms with 0.05% AsF_3 or 0.05% PF_3 in solid neon.[9] **C**: F_2B–As=BF/F_2B–P=BF. Unidentified bands are marked with asterisks.

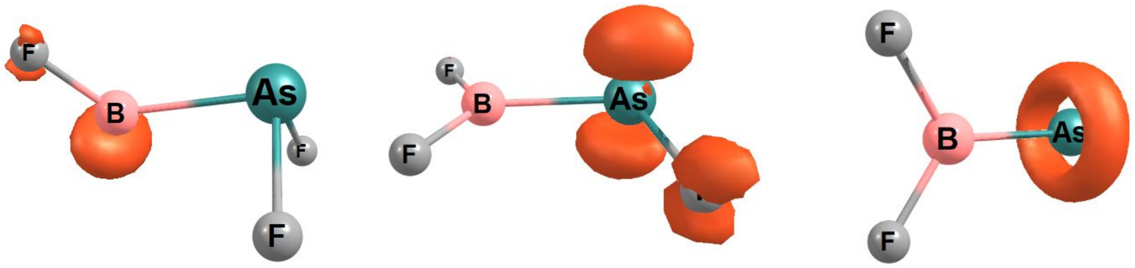

$FBAsF_2$ (C_1, ^2A) F_2BAsF (C_s, $^2A''$) F_2BAs (C_{2v}, $^3A_2$)

Figure S8. Spin-density isosurfaces of FB–AsF_2, F_2B–AsF and F_2B–As at 0.03 Å^{-3} calculated at the B3LYP/def2-TZVPPD level of theory.

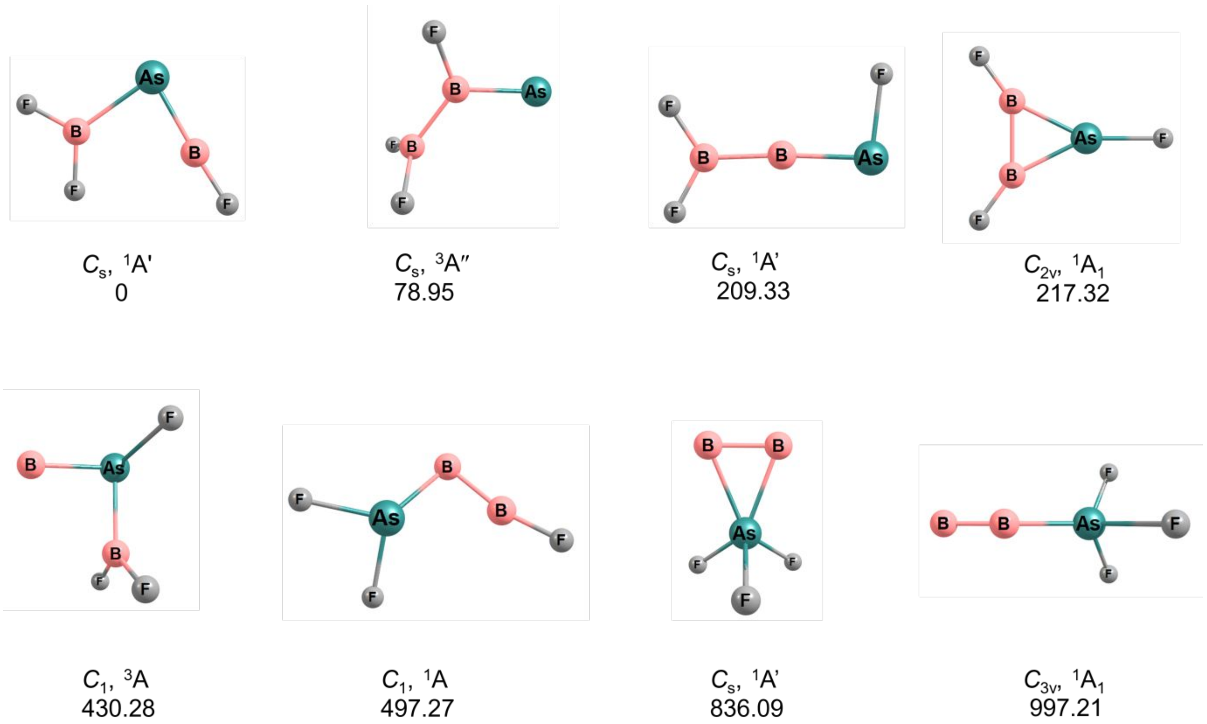

Figure S9. Isomers of the formula B_2AsF_3 with relative stabilities (electronic energies + ZPE correction) in kJ mol^{-1} calculated at B3LYP/def2-TZVPPD level of theory.

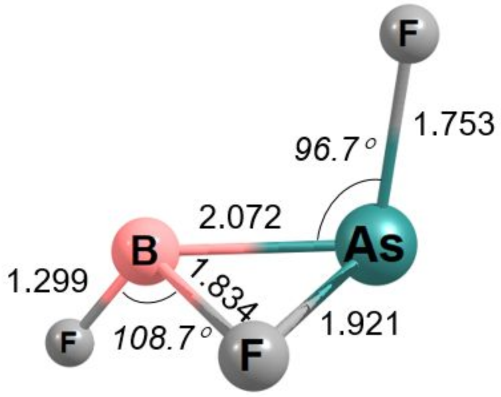

TS1-D (C_1, ^2A)

Frequency	Intensity
-306.4	–
96.2	4
174.6	5
257.1	2
334.1	1
419.3	25
542.9	82
657.3	101
1322.7	404

Figure S10. Transition state structure (TS1-D) for the for the mutual isomerization of doublet FB–AsF_2 to F_2B–AsF (Scheme S1) and its vibrational wavenumbers (cm^{-1}) calculated at the B3LYP/def2-TZVPPD level of theory.

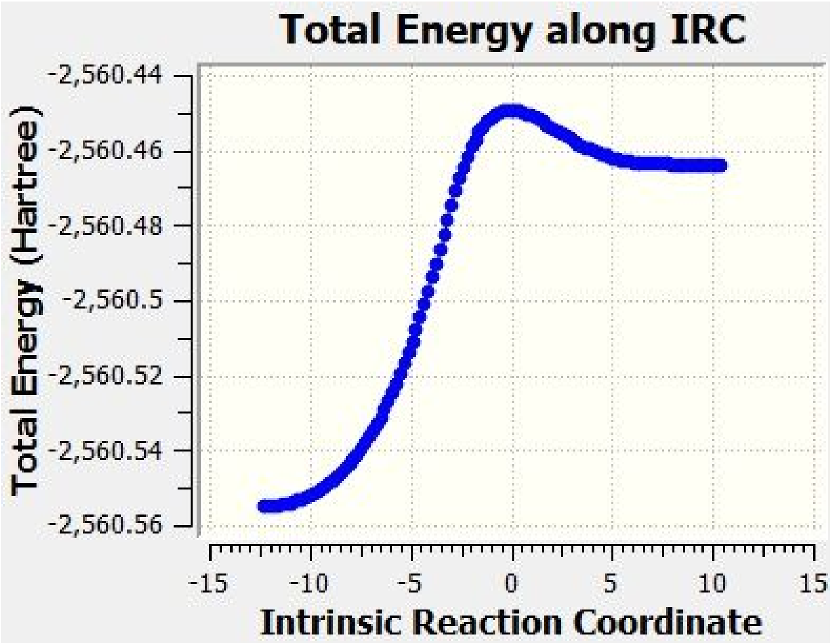

Figure S11. Intrinsic reaction coordinate (IRC) via the transition state TS1-D (Scheme S1) for the mutual isomerization of doublet FB–AsF_2 to F_2B–AsF (lower in energy) obtained at the B3LYP/def2-TZVPPD level of theory.

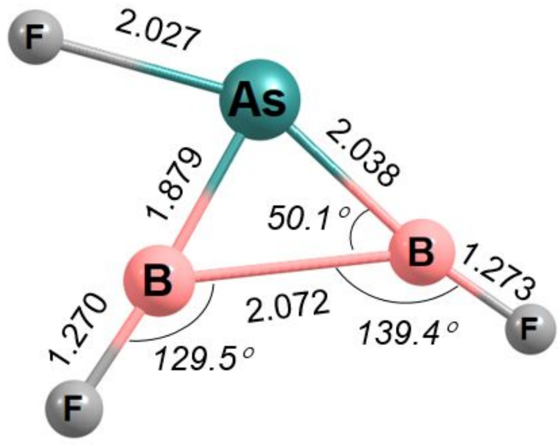

TS2 (C_s, $^1A'$)

Frequency	Intensity
-287.4	–
8.8	4
41.9	2
263.4	11
314.1	1
316.5	4
407.2	166
439.7	1
512.9	5
1416.4	492
1534.8	441

Figure S12. Transition state structure (TS2-S) for the mutual isomerization of singlet cyclic FB–(AsF)–BF to F_2B–As=BF (Scheme S1) and its wavenumbers (cm^{-1}) calculated at the B3LYP/def2-TZVPPD level of theory.

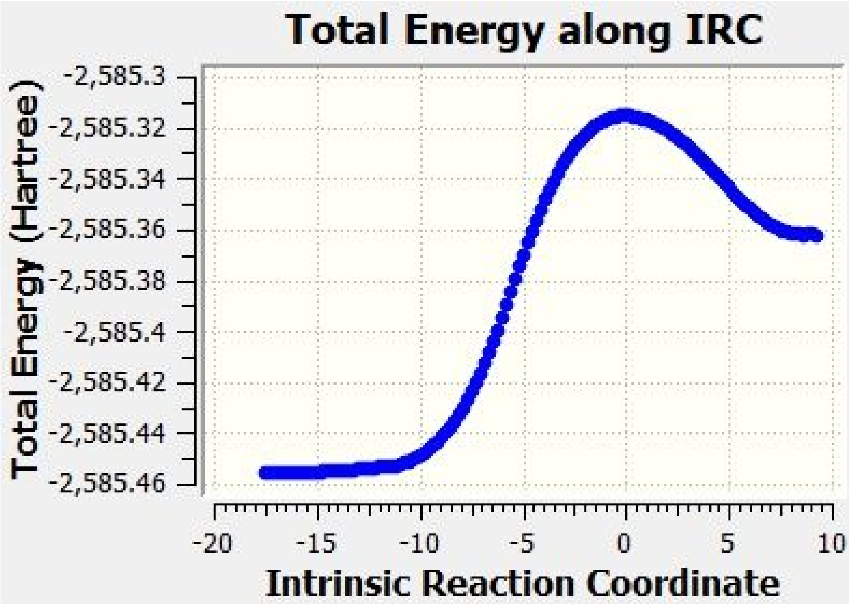

Figure S13. Intrinsic reaction coordinate (IRC) via the transition state TS2-S (Scheme S1) for the mutual isomerization of singlet cyclic-FB–(AsF)–BF to F_2B–As=BF (lower in energy) obtained at the B3LYP/def2-TZVPPD level of theory.

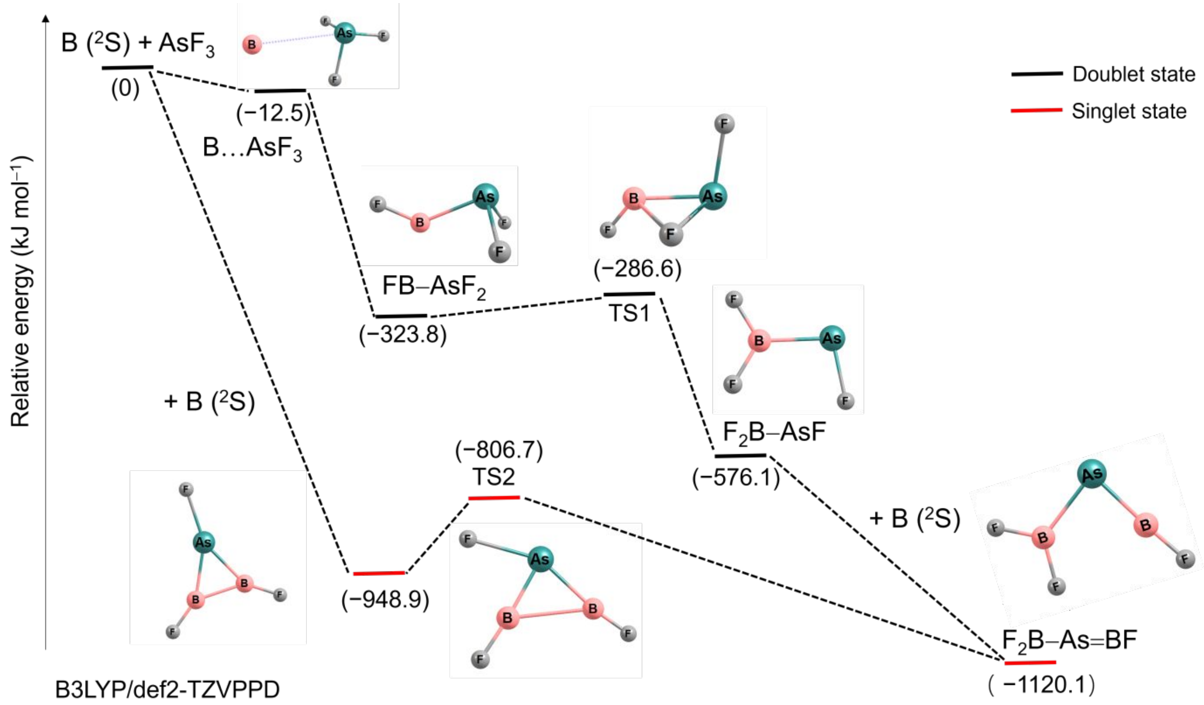

Scheme S1. Potential energy surface (electronic energies + ZPE correction) in kJ mol^{-1} for species formed from laser ablated boron atoms with AsF_3 obtained at the B3LYP/def2-TZVPPD level of theory.

Calculated atomic coordinates (in Å) and total energies of species for optimized structures at the B3LYP/def2-TZVPPD level.

$BAsF_3$ $^4A_1$ (C_{3v})

Energy = -2560.296012

F	0.00000000	1.47967700	-0.65082000
F	-1.28143800	-0.73983900	-0.65082000
F	1.28143800	-0.73983900	-0.65082000
B	0.00000000	0.00000000	2.09028700
As	0.00000000	0.00000000	0.21577900

$BAsF_3$ $^2A'$ (C_s)

Energy = -2560.336264

F	0.75916500	-0.08425800	1.30011000
F	-0.46256600	1.73494600	0.00000000
F	0.75916500	-0.08425800	-1.30011000
B	0.75916500	-2.74333000	0.00000000
As	-0.40296000	-0.01155200	0.00000000

FB–AsF_2 ^2A (C_1)

Energy = -2560.454783

F	0.65760700	1.43855300	0.55882000
F	1.27844500	-1.13076000	0.61316100
F	-2.62463200	-0.07654700	0.01789700
B	-1.49394200	-0.37212800	0.57696800
As	0.41414900	-0.00668400	-0.41193200

F_2B–AsF $^2A''$ (C_s)

Energy = -2560.551006

F	-0.35760700	-2.05411800	0.00000000
F	1.81065200	-1.38832500	0.00000000
F	-1.74343900	0.65467600	0.00000000
B	……0.52271000	-1.07590500	0.00000000
As	0.00000000	0.92331600	0.00000000

Cyclic-FB–(AsF)–BF $^1A_1$ (C_{2v})

Energy = -2585.357001

F	0.00000000	0.00000000	2.44784500
F	0.00000000	1.91163200	-1.83714600
F	0.00000000	-1.91163200	-1.83714600
B	0.00000000	0.86292400	-1.07010600
B	0.00000000	-0.86292400	-1.07010600
As	0.00000000	0.00000000	0.65876000

F_2B–As=BF $^1A'$ (C_s)

Energy = -2585.439566

F	-0.50185300	-1.95472600	0.00000000
F	-2.35011900	-0.66598800	0.00000000
F	2.58203600	-0.73431000	0.00000000
B	-1.02612300	-0.72847800	0.00000000
B	1.51200700	-0.04470200	0.00000000

As	0.00000000	1.03215500	0.00000000

TS1 ^{2}A (C_1)

Energy = -2560.440641

F	-1.60722200	-0.68475500	0.77913400
F	0.63277800	1.15540500	0.63432300
F	2.43340200	-0.51211800	-0.11194600
B	1.22453400	-0.55881300	0.36231700
As	-0.58343300	0.09597800	-0.40985400

TS2 ^{2}A' (C_S)

Energy = -2585.302663

F	0.53822000	2.39472600	0.00082200
F	-2.95017900	0.07695500	-0.00291600
As	0.25003100	-0.73840700	0.00137600
B	0.36282300	1.13712500	0.00108700
B	-1.69335700	-0.12397800	0.00118300
F	2.23436300	-0.32704700	-0.00421300

F_2BAs $^3A_2$ (C_{2v})

Energy = -2460.623938

F	0.00000000	1.12777700	-1.68342600
F	0.00000000	-1.12777700	-1.68342600
B	0.00000000	0.00000000	-0.98917300
As	0.00000000	0.00000000	1.06810700

Calculated atomic coordinates (in Å) of species for optimized structures at the CCSD(T)/def2-TZVPPD level.

$BAsF_3$ $^4A_1$ (C_{3v})
Energy = -2558.226215

F	1.466371457	0.000000000	0.658556649
F	-0.733181822	-1.269900703	0.658546504
F	-0.733181822	1.269900703	0.658546504
B	-0.000004370	0.000000000	-2.072605088
As	-0.000003441	0.000000000	-0.196425464

$BAsF_3$ $^2A'$ (C_s)
Energy = -2558.259168

F	0.740044473	-0.041740322	1.276047647
F	-0.465809885	1.784477599	0.000000000
F	0.740044473	-0.041740322	-1.276047647
B	0.811235464	-2.950816848	0.000000000
As	-0.413545524	0.061367891	0.000000000

FB–AsF_2 ^2A (C_1)
Energy = -2558.365466

F	-0.801363954	1.428928563	-0.348452481
F	-1.498283805	-1.084738363	-0.357552523
F	2.450304070	-0.098120032	0.005119918
B	1.282623462	-0.430917758	-0.461629258
As	-0.546966709	-0.004693424	0.614949306

F_2B–AsF $^2A''$ (C_s)
Energy = -2558.463227

F	-0.364820783	-2.042432436	0.000000000
F	1.808799908	-1.392103893	0.000000000
F	-1.733830099	0.637435783	0.000000000
B	0.523519286	-1.070815792	0.000000000
As	-0.001352294	0.927560127	0.000000000

Cyclic-FB–(AsF)–BF $^1A_1$ (C_{2v})
Energy = -2583.200349

F	0.000000000	0.000000000	2.438068370
F	0.000000000	1.922178799	-1.835886665
F	0.000000000	-1.922178799	-1.835886665
B	0.000000000	0.867216032	-1.071200644
B	0.000000000	-0.867216032	-1.071200644
As	0.000000000	0.000000000	0.668207052

F_2B–As=BF $^1A'$ (C_s)
Energy = -2583.283408

F	-0.432418584	-1.922472619	0.000000000
F	-2.322355326	-0.695667291	0.000000000
F	2.509503520	-0.794879043	0.000000000
B	-0.997104264	-0.711065856	0.000000000
B	1.471255408	-0.052062160	0.000000000

As	-0.012932739	1.080097746	0.000000000

F_2B–As $^3A_2$ (C_{2v})

Energy = -2458.685873

F	0.000000000	1.128027982	-1.684609641
F	0.000000000	-1.128027982	-1.684609641
B	0.000000000	0.000000000	-0.991426739
As	0.000000000	0.000000000	1.072727784
